# Gendered Dimensions of Social Support in Therapeutic Communities: A Qualitative Study on Recovery Pathways Among Individuals with Substance Use Disorders in Brazil

**DOI:** 10.1192/j.eurpsy.2026.10898

**Published:** 2026-07-31

**Authors:** R. A. Bosso, M. A. D. Santos, A. Diehl, A. R. D. Almeida, A. L. Granjeiro, J. D. O. Bertholini, V. D. Santos, S. C. Pillon

**Affiliations:** 1 Psiquiatria; 2 Psicologia, Universidade de São Paulo - USP, Ribeirão Preto; 3 Psiquiatria, ABEAD, Londrina; 4 Psiquiatria, Unicamp; 5 Psicologia, Pesquisador Autônomo, Campinas, Brazil; 6 Psicologia, Fundação Universitária Iberoamericana - FUNIBER, Barcelona, Spain; 7 Psicologia, Faculdade Anhanguera , Campinas, Brazil

## Abstract

**Introduction:**

Social support plays a central role in the recovery of individuals with substance use disorders (SUDs). It not only enhances treatment adherence but also helps sustain connections, reinforce motivation, and foster a sense of belonging. Yet, the ways in which gender norms influence the reception and provision of support within therapeutic communities (TCs) in Brazil remain under-explored.

**Objectives:**

To understand how individuals with SUDs perceive and experience social support during treatment in TCs, considering gender influences and the role of family and community ties in their care trajectories.

**Methods:**

This is a qualitative exploratory study. A focus group was conducted with six participants (three men, including one trans man, and three women, including one trans woman) in a TC in an inland region of São Paulo state, Brazil. Discussions were audio-recorded, transcribed, and subjected to thematic content analysis.

**Results:**

Three primary themes emerged: (1) **Emotional and instrumental support:** Affectionate encouragement, attentive listening, and everyday presence were valued as fundamental, while lack of understanding by family members weakened support; (2) **Family and institutional networks:** When family ties were fragile, bonds forged in the TC and in social assistance networks assumed a central role in maintaining treatment motivation; (3) **Gender norms:** Men reported difficulties expressing vulnerability and seeking help, whereas women emphasized the importance of affective ties and acceptance from family and peers. These differences affected how support was accessed and incorporated into recovery pathways.

**Conclusions:**

In TCs, social support emerges at the intersection of affective, institutional, and gendered dimensions. Recognizing the influence of gender norms is essential to foster equitable access to emotional support. We argue that policies and practices should strengthen family and institutional networks while creating environments that validate vulnerability and emotional expression among men, women, and trans people in treatment.

**Disclosure of Interest:**

None Declared

